# Effect of Light-Sources and Thicknesses of Composite Onlays on Micro-Hardness of Luting Composites

**DOI:** 10.3390/ma14226849

**Published:** 2021-11-13

**Authors:** Francesco De Angelis, Mirco Vadini, Mario Capogreco, Camillo D’Arcangelo, Maurizio D’Amario

**Affiliations:** 1 Unit of Restorative Dentistry and Endodontics, Department of Medical, Oral and Biotechnological Sciences, Dental School, “G. D’Annunzio” University of Chieti, 66100 Chieti, Italy; fda580@gmail.com (F.D.A.); cdarcang@unich.it (C.D.); 2 Unit of Restorative Dentistry, Endodontics and Oral Pathology, Dental Clinic, Department of Life, Health and Environmental Sciences, University of L’Aquila, 67100 L’Aquila, Italy; mario.capogreco@univaq.it (M.C.); maurizio.damario@univaq.it (M.D.)

**Keywords:** cementation, hardness, indirect restoration, light-curing units, luting, resin-based luting cements

## Abstract

The aim of this study was to compare three different light-curing-units (LCUs) and determine their effectiveness in the adhesive cementation of indirect composite restorations when a light-curing resin cement is used. Two resin composites were selected: Enamel Plus HRI (Micerium) and AURA (SDI). Three thicknesses (3 mm, 4 mm and 5 mm) were produced and applied as overlays and underlays for each resin composite. A standardized composite layer was placed between underlay and overlay surfaces. Light curing of the resin-based luting composites was attained through the overlay filters using LCUs for different exposure times. All specimens were allocated to experimental groups according to the overlay thickness, curing unit and curing time. Vickers Hardness (VH) notches were carried out on each specimen. Data were statistically evaluated. The curing unit, curing time and overlay thickness were significant factors capable of influencing VH values. The results showed significantly decreased VH values with increasing specimen thickness (*p* < 0.05). Significant differences in VH values were found amongst the LCUs for the various exposure times (*p* < 0.05). According to the results, a time of cure shorter than 80 s (with a conventional quartz–tungsten–halogen LCU) or shorter than 40 s (with a high-power light-emitting diode (LED) LCU) is not recommended. The only subgroup achieving clinically acceptable VH values after a short 20 s curing time included the 3 mm-thick overlays made out of the AURA composite, when the high-power LED LCU unit was used (VH 51.0). Composite thickness has an intense effect on polymerization. In clinical practice, light-cured resin cements may result in insufficient polymerization for high thickness and inadequate times. High-intensity curing lights can attain the sufficient polymerization of resin cements through overlays in a significantly shorter time than conventional halogen light.

## 1. Introduction

Several types of resin-based luting composites (RBLCs) are currently used to bond indirect restorations. RBLCs may be classified based on their adhesive features (etch-and-rinse, self-etch and self-adhesive systems) and their polymerization initiation mode (self-curable, light-curable and dual-curable) [1]. Traditionally, for the placement of indirect restorations, a dual-curable RBLC is adopted to safeguard effective cement polymerization. On the other hand, light-curable RBLCs have several advantages: improved handling (formulated as a single paste that does not require mixing), extended working time, simplification of surplus cement elimination procedures and, as a consequence, an easier full sitting of the restoration [2]. Moreover, light-curable RBLCs generally contain an increased amount of filler than traditional dual-cure RBLCs, enabling higher intrinsic mechanical properties [3]. Although a higher filler load also means reduced fluidity, a clinically ideal viscosity can still be achieved by means of appropriate preheating procedures [4,5].

All these clear advantages justified the attempt to extend the employment of cements based on light-curable chemistries beyond the limits of laminate veneers [6,7] or thin inlays [8,9], and also suggested a better investigation of their effectiveness when dealing with thicker posterior restorations, such as overlays or endocrowns. In fact, it must be underlined that light-curable RBLCs cannot be considered as indiscriminately suitable for every circumstance, and their use should be prudently limited to clinical situations in which a sufficient irradiance of the luting agent can be guaranteed. An adequate polymerization of RBLCs underneath any bonded restoration is always required, as the degree of monomer conversion of the resin is closely related to its final mechanical and biological properties [10,11]. Inadequate polymerization may compromise the cement’s physical–mechanical properties, leading to increased solubility, dimensional instability, color change and reduced biocompatibility [12], which may consequently affect the restoration’s longevity and ultimate clinical success [13,14]. Previous research has clearly shown how excessively thick and/or opaque restorations should suggest the selection of a dual-curing luting agent.

Light polymerization in resin-based materials may be achieved by means of different kinds of light-curing units (LCUs). Until recently, conventional quartz–tungsten–halogen (QTH) LCUs were the most used. However, their usage diminished due to their inherent disadvantages, such as halogen bulbs having a restricted effective lifetime. In recent years, light-emitting diodes (LED) have also become accessible. LEDs with a lifetime span of over 10,000 h show little degradation of light output over this time, a clear advantage when compared with halogen bulbs. Furthermore, LEDs require no filters to generate blue light, are very resistant to shock and vibration and are suitable for portable use considering their relatively low power consumption [15,16].

Thus far, few studies have examined the effectiveness of purely light-curable composites to bond indirect restorations [2,3,17,18]. In particular, insufficient data are available on the effect of different light-curing units (LCUs) on the specific success of any particular luting protocol. Therefore, the aim of this study was to evaluate the Vickers micro-hardness (VH) of two light-curable RBLCs used for the adhesive cementation of indirect composite restorations, following their irradiance for different curing times, by means of different LCUs and across composite overlays with varying thicknesses. The null hypotheses tested were that the resin cement micro-hardness would not be influenced by the curing time, the curing unit or the overlay thickness.

## 2. Materials and Methods

Two commercially available resin composites were selected for this study: Enamel Plus HRI (UD3 shade—Micerium; Avegno, Genova, Italy) and AURA (MC3 shade—SDI; Bayswater, Australia) (Table 1). 

Specimen preparation for hardness measurements was performed as previously proposed [2,19].

### 2.1. Resin Composite Overlays and Underlays Production

Composite pastes were positioned into cylindrical molds with a 10 mm inner diameter. Layering was carried out in five increments of approximately 2 mm each, which were individually light polymerized for 40 s (Bluephase C8; Ivoclar Vivadent AG, Schaan, Liechtenstein, with 800 mW/cm^2^ output). After having removed the molds, the 10 mm high composite cylinders were subjected to an additional cycle of polymerization in a composite oven at 70 °C for 10 min (Bulb PlusT; Micerium). In order to obtain disks with perfectly flat and parallel circular bases, cured cylinders were locked on the arm of a Micromet M machine (Remet, Casalecchio di Reno, Bologna, Italy) and subjected to consecutive cuts perpendicularly to their long axis. The distance between the consecutive cuts was regulated to produce cylinders with 3 different thicknesses: 3 mm, 4 mm and 5 mm. The thickness of each cylinder was controlled using a digital caliper (series 500 Caliper; Mitutoyo America Corp, Aurora, IL, USA) with an accuracy of 0.01 mm.

**Figure 1:**
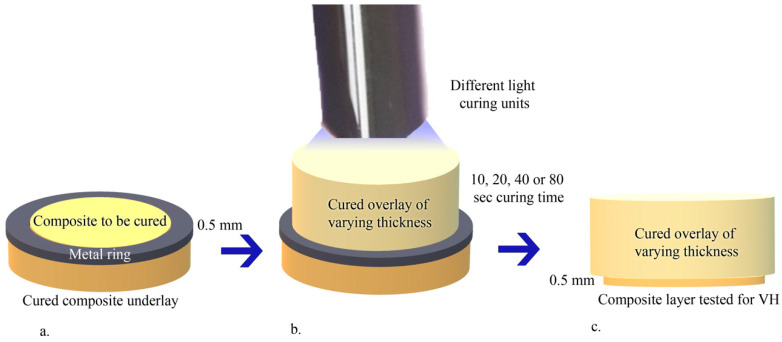
Specimen preparation (**a**,**b**) and final specimen shape (**c**). The 0.5 mm-thick resin layer was subjected to the Vickers Hardness evaluation.

For each of the 2 resin composites under investigation, 108 cylinders to be used as overlays for every different thickness were produced, resulting in a total of 324. Further cylinders with 2 mm fixed thicknesses were similarly prepared and served as underlays (Figure 1). 

Additional heat-cured, 2 mm-thick specimens, 9 for each resin composite under investigation, were produced, assigned to control groups and used to establish a hardness reference value for the tested materials.

### 2.2. Experimental Group Specimen Production

One surface of each overlay was subjected to air-borne particle abrasion with 50 μm Al2O3 (Korox; Bego Bremen, Germany) using an intraoral air-abrasion device (Dento-Prep; Micerium); the tip of the micro-etcher was kept 5 cm away from the surface and applied for 10 s at 2.0 bar pressure [9]. The surfaces were then rinsed with water and thoroughly dried. A standardized amount of composite paste was placed between the underlay and the overlay bonding surface under low lighting conditions to inhibit photo-activation. A 0.5 mm-thick metal ring, with a 7 mm diameter inner hole, was maintained between the underlay and the overlay in order to keep them at a fixed distance and, thus, to achieve a standard thickness of the composite to be cured. A 0.05 mm-thick transparent polyethylene strip between the underlay and the uncured composite was used to avoid bonding at this interface. For each specimen, the 0.5 mm composite layer to be cured, the cured overlay and the underlay were made out of the same batch of resin composite. The specimens, which at this point were assigned to two experimental groups on the basis of the resin composite used (Enamel Plus HRI: ENA group; AURA: AUR group) and to subgroups on the basis of the overlay thickness (3, 4, 5 mm), were further divided into extra subgroups on the basis of the three different LCUs employed (Table 2) and the curing time, which was carried out for 10, 20, 40 or 80 s, resulting in a total of 72 experimental subgroups, each composed of 9 specimens (n = 9). This particular sample size was considered appropriate, based on the sample size adopted in similar previous studies [2].

The light intensity was monitored with an LED Curing Light Meter radiometer (LM-1; Guilin Woodpecker Medical Instrument Co Ltd., Guilin, China) throughout the experiment, with measurements being taken before and after the use of the light-curing unit. Polymerization was performed by placing the selected curing unit tip directly in contact with the central part of the overlay upper surface. In this way, the underlays served as reflective material, while the cured overlays of varying thickness were used to control the amount of light reaching the composite to be cured and, thus, its micro-hardness. Obtained specimens, whose shape is depicted in Figure 1, were stored at room temperature in black film canisters before subsequent procedures.

### 2.3. Vickers Hardness Measurement

In the experimental groups, Vickers hardness (VH) readings were recorded on the central part of the lower free surface of the 0.5 mm-thick composite layer that was light-cured through the overlay (Figure 1). In the control groups, the micro-hardness of the heat-cured specimens was determined and assumed as the optimum micro-hardness (OM) for each respective material. This was performed whilst considering that heat-curing leads both to the highest degree of conversion and to the best mechanical properties, including micro-hardness [20,21].

**Figure 2:**
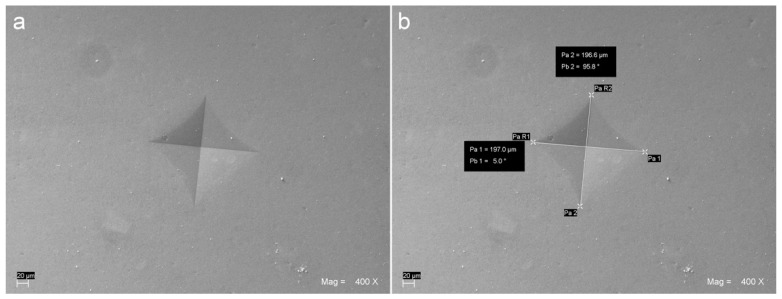
Scanning electron micro-graph showing a VH indentation (**a**) and the measurement of its diagonals (**b**) performed on a specimen from ENA group, 3 mm thick and cured for 40 s using a halogen curing unit with 600 mW/cm^2^ output.

**Figure 3:**
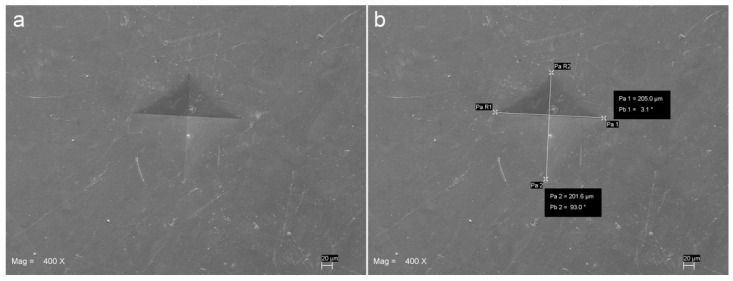
Scanning electron micro-graph showing the VH indentation (**a**) and the measurement of its diagonals (**b**) performed on a specimen from AUR group, 3 mm thick and cured for 10 s using a LED curing unit with 1500 mW/cm^2^ output.

For each specimen, the mean value of the three VH readings performed at approximately 2 mm distance from one another was used as the raw datum. Vickers indentation was produced by applying a 10 N load for 10 s using a Universal Testing Machine with a 500 N load cell (Lloyd LR 30 K—Lloyd Instruments Ltd.; Fareham, UK) provided with a standard 136° Vickers diamond indenter (item #17; Affri, Induno Olona, Varese, Italy). Scanning electron micro-photographs (EVO 50 XVP LaB6; Carl Zeiss SMT Ltd., Cambridge, UK) were taken at different magnifications in order to measure the linear extent of the indentation diagonals (Figure 2 and Figure 3). 

Then, VH numbers were calculated according to the following formula:

VH = (1.854 ∗ F)/[(d_1_ + d_2_)/2]^2,^

where d_1_ and d_2_ are the measured diagonals (mm), and F is the predetermined applied load expressed in kilograms/force (1.0204 Kg).

### 2.4. Data Analysis

Raw data achieved in the experimental groups were split, based on the two resin composites, and then arranged on the basis of the three factors under investigation (curing time, overlay thickness and LCU). Means and standard deviations were calculated. After having checked for the homogeneity of variances (Levene’s test) and that data were normally distributed (Kolmogorov–Smirnov test), the effect of the three factors on the mean VH values within (and not between) the two composite materials was analyzed using three-way ANOVA tests. Multiple comparisons were performed according to the Holm–Sidak method. Values of *p* < 0.05 were considered statistically significant in all tests.

The mean OM values were also calculated for each composite in the two control groups. It has been reported that the curing extent of a composite layer at the bottom of a deep cavity is considered to be acceptable if its hardness is above 80% of the maximum hardness value measured on the specimen surface [16,22]. As a consequence, VH values below 80% of the OM value recorded on corresponding heat-cured specimens were not considered clinically acceptable.

## 3. Results

The results of the three-way analysis of variance displayed that, with both composites, the mean VH values were statistically influenced by the curing time, the overlay thickness and the LCU (*p* < 0.05) (Table 3). Thus, the null hypotheses tested were all rejected.

**Figure 4:**
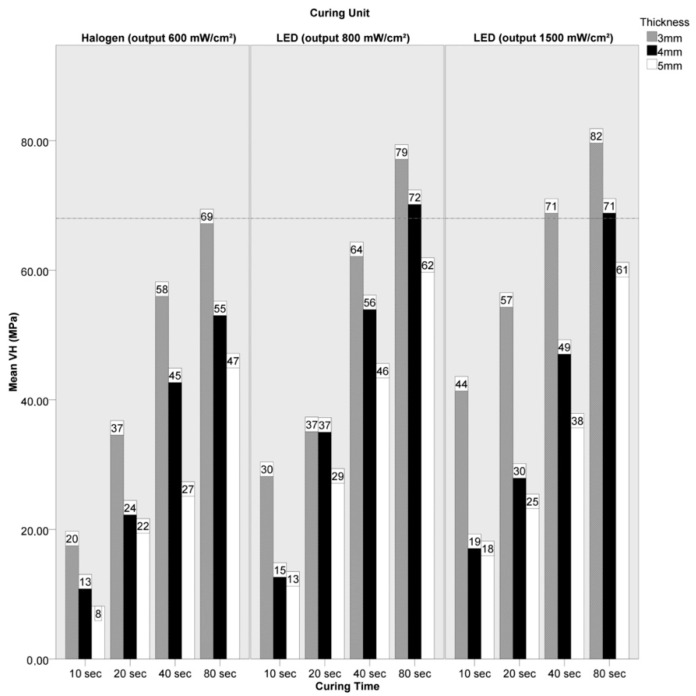
Bar chart summarizing mean Vickers Hardness (VH) numbers obtained in the ENA group using overlays of different thicknesses and after 10, 20, 40 or 80 s curing time. The horizontal line indicates 80% of the VH value achieved in the respective control group, which was used as the acceptability threshold for this material.

**Figure 5:**
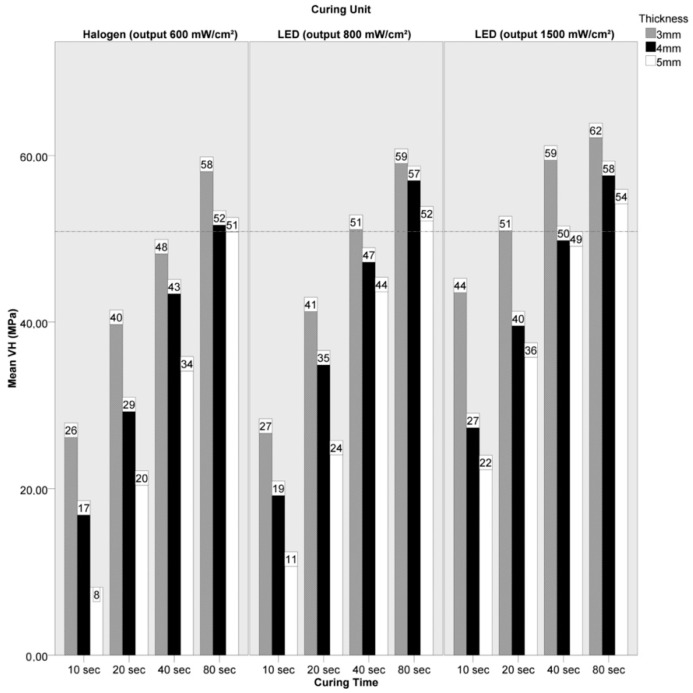
Bar chart summarizing mean Vickers Hardness (VH) numbers obtained in the AUR group using overlays with different thicknesses and after 10, 20, 40 or 80 s curing time. The horizontal line indicates 80% of the VH value achieved in the respective control group, which was used as the acceptability threshold for this material.

The mean VH values achieved in the experimental groups and the reference OM values recorded in the control groups are summarized in bar charts (Figure 4 and Figure 5) and in Table 4, where the standard deviations and the Holm–Sidak test results are also given.

In the ENA group, clinically acceptable hardness values (at least 80% of the control) were achieved after 80 s curing time using 3 mm overlays, when the Demetron LC (600 mW/cm^2^ output) unit was used (VH 69.4); after 80 s using 4 mm or thinner overlays, when the Bluephase C8 (800 mW/cm^2^ output) unit was used (VH 72.4 and 79.4, respectively) and when the Starlight Uno (1500 mW/cm^2^ output) unit was used (VH 71.0 and 82.3, respectively); and after 40 s using 3 mm overlays when the Starlight Uno (1500 mW/cm^2^ output) unit was used (VH 71.0).

In the AUR group, clinically acceptable hardness values were achieved after 80 s curing time using 5, 4 and 3 mm overlays, when the Demetron LC (600 mW/cm^2^ output) unit was used (VH 50.8, 51.6 ad 58.1, respectively), when the Bluephase C8 (800 mW/cm^2^ output) unit was used (VH 52.1, 57.0 and 59.1, respectively) and when the Starlight Uno (1500 mW/cm^2^ output) unit was used (VH 54.5, 57.3 and 62.6, respectively); after 40 s using 3 mm overlays, when the Bluephase C8 (800 mW/cm^2^ output) unit was used (VH 51.1); after 40 s using 4 mm or thinner overlays, when the Starlight Uno (1500 mW/cm^2^ output) unit was used (VH 49.8 and 59.4, respectively); and after 20 s using 3 mm overlays, when the Starlight Uno (1500 mW/cm^2^ output) unit was used (VH 51.0).

## 4. Discussion

The null hypotheses of the present study had to be rejected, considering that all the variables under investigation (LCU, curing time and overlay thickness) had significant effects on the VH of the tested specimens. The study confirmed that resin composite overlays can significantly attenuate the light from the LCU, even for thicknesses that may commonly occur in a clinical setting [2]. The results showed significantly decreased VH values with increasing specimen thickness (*p* < 0.05). This validates the observations of other studies [2,17,23,24], in which light curing through composite or ceramic as compared with direct irradiation decreased the values for many mechanical parameters of materials. The weakening of the curing light by passing through the overlay discs may reduce the light intensity and consequently decrease the degree of polymerization of RBLCs. The curing-light weakening is firmly associated with the properties of the restorative material, particularly due to its thickness, opacity and shade [25,26]. As a consequence, an adequate light polymerization of all portions of the light-cured cement appears not always possible. An extended curing time would be required as the thickness of the composite overlay rises, and beyond a certain limit, light-cured RBLCs may not be used as luting materials. According to the results of this study, a time of cure shorter than 80 s (if a QTH LCU is used) or shorter than 40 s (if a high-power LED LCU is used) is not recommended. The only subgroup achieving clinically acceptable VH values after a short 20 s curing time included the 3 mm-thick overlays made out of AUR composite, when the high-power LED LCU (Starlight Uno) unit was used (VH 51.0).

The process of polymerization begins when the light from the light-curing unit activates the photoinitiator [27]. Camphorquinone is the primer photoinitiator of the light-cured RBLCs. Incomplete polymerization of the composite material will affect its physical properties, along with surface VH. Additionally, the wear properties and biocompatibility of dental composites can be affected by the efficiency of polymerization [28,29]. In the current study, resin cement was arranged in 0.5 mm thicknesses to simulate clinical conditions, and in order to allow an accurate measurement of its surface hardness by means of Vickers test. Surface VH is a significant parameter for evaluating the physical properties of dental materials, and it is described as the resistance of a material to indentation or penetration [26]. In the literature, resin hardness is strongly related to its conversion degree because the higher the conversion degree, the greater the number of cross-linked polymers and consequently the hardness of the material [30,31]. This is the reason for the frequent use of VH as an easy and reliable method for indirectly evaluating the conversion degree of resin-based cements [2,13,30].

In 2012, D’Arcangelo et al. [2] advised clinicians to carefully control overlay thickness by conveniently modifying the cavity shape with a direct resin composite build-up. Moreover, they suggested the need to investigate other important variables, such as the actual power density of the LCUs. In fact, since the indirect restoration can absorb, reflect and refract the curing light, the extent of total energy from the light source reaching the cement should be considered in order to accomplish adequate polymerization of resin cements [32,33]. In 2015, Cho et al. [34] demonstrated a gradual decrease in light intensity from the 0 mm (control) (900 mW/cm^2^) to the 0.3 mm ceramic thickness (585 mW/cm^2^) and to the 1.2 mm thickness (549 mW/cm^2^). Disparate recommendations have been made to address the issue of light attenuation. Longer polymerization times and multidirectional curing were proposed to overcome the effect of material thickness [2,35]. Regarding the relationship between the type of light-curing unit and light-curing time, there are adverse accounts in the literature [32,33]. QTH lamps were once the most widely used light-curing units, and in some place they still are. They emit a continuous spectrum, and a large amount of the total energy produced by a halogen light is converted to heat, which needs to be dispersed by means of a small fan incorporated in the device. No more than 0.5% of the wavelength produced is effective for curing [36,37]. More drawbacks are a deterioration of irradiance over time [38] and a limited extent of cure. On the contrary, light-emitting diode (LED) lights provide a much narrower emission spectrum (around 470 nm, with a bandwidth of about 20 nm), which is close to the absorption range of camphorquinone. In general, the LED light possesses the advantages of extended lifetimes, limited degradation of light output over time, prevention of over-heating and resistance to shock and vibration [39]. The latest LED lights with high-power irradiance and suggested shorter exposure times have been introduced to the dental market; however, the efficiency of the latest generations of LED lights in the polymerization of various RBLCs under indirect restorations has not yet been fully investigated.

The criterion that manufacturers generally utilize to describe a curing light is its tip irradiance (radiant exitance), expressed as the power per unit area (mW/cm^2^). In the present study, one QTH and two LED LCUs (with 600, 800 and 1500 mW/cm^2^ output, respectively) were included. High-power LCUs provide a higher energy density in a shorter period. The depth of cure is, however, likely the same, despite such a reduced irradiation time. In this study, the satisfactory level of polymerization was defined as the polymerization extent at which the surface hardness was at least 80% of the maximum surface hardness measured on post-cured samples in the control groups, based on results reported in previous papers [16,22]. To limit any additional variability related to the light-curing procedure, the tips of the light-curing units were always maintained in the center of the overlays. Further to this point, it is important to consider that the diameter of the 0.5 mm-thick composite layer to be cured (7 mm) was less than that of the smallest LCU tip employed herein (8 mm, for Starlight Uno). In addition, the three VH indentations were carefully made in the central part of every specimen, which is the area receiving the maximum possible irradiance.

Among the possible limits of the present study, it must be underlined how the polymerization kinetics of composites, together with their mechanical properties, are highly dependent on the features of their filler particles, such as size, composition and content [40,41]. Smaller filler particles (hence, a larger surface area) and higher filler loading seem to lead to increased composite viscosity, which cause the polymerization rates to be slowed down [42]. Indeed, the literature also suggests that the type of base monomer included in composite formulation can greatly influence the physical–mechanical properties [43]. Considering the different compositions of the two tested composites, a difference regarding polymerization kinetics could be expected. However, in the present study, statistical comparisons were only performed within (not between) the two composite materials, in order to limit the number of variables and to avoid further complication of an already complex three-factor model. Moreover, a more thorough analysis of the light-curable RBLC layer, identifying the presence of unreactive monomers, for a direct determination of its actual degree of conversion (by means of Fourier transform infrared spectroscopy or Raman spectroscopy) and/or for the assessment of other paramount mechanical properties, would represent an interesting subject for further studies, with the aim of strengthening the VH results of the present investigation.

## 5. Conclusions

Despite the limitations of the present in vitro study, it can be concluded that all three investigated light sources were efficient, but light transmission through composite overlays seems to be more efficient using a high-power LCU. The VH values of the tested resin composites used as luting agents were affected by all the tested variables (curing unit, curing time and restoration thickness). The clinical implication is that the thicker the composite overlay, the greater the exposure time and the energy density delivered on each face of the restoration need to be, with the aim to provide enough power for appropriate polymerization of the underlying resin cement. With at least 80 s curing time, a 4/5 mm thickness limit should not be exceeded, depending on the overlay material, if a light-curing composite has to be used for cementation.

## Figures and Tables

**Table 1:** Summary of the materials used in experimental group.

Group	Resin Composite	Resin Matrix	Filler	Content % (*w*/*v*)	Manufacturer
ENA	Enamel Plus HRI (UD3 shade) (Batch n. 2014001144)	BisGMA, UDMA, TEGMA	Glass filler, SiO_2_	75/53	Micerium S.p.A. (Avegno, Genova, Italy)
AUR	AURA SDI (MC3 shade) (Batch n. 130810)	BisGMA, UDMA, BisEMA	Ba–Al–B–Si glass, Ba glass	78/63	SDI Limited (Bayswater, Australia)

**Table 2:** Characteristics of the employed LCUs.

LCU	LCU Type	Output (mW/cm^2^)	Tip Diameter (mm)	Manufacturer
Demetron LC	QTH	600	7.9	Kerr Corporation, (Orange, CA, USA)
Bluephase C8	LED	800	9.8	Ivoclar Vivadent AG (Schaan, Liechtenstein)
Starlight Uno	LED	1500	8.0	Mectron S.p.A. (Genova, Italy)

**Table 3:** Three-way ANOVA tables showing effects of the three variables (curing time, overlay thickness and LCU) on the Vickers Hardness mean values.

**ENA Group**					
**Factor**	**Degrees of Freedom**	**Sum of Squares**	**Mean Squares**	**F**	***p***
Curing Time	3	100,050.491	33,350.164	853.147	<0.001
Overlay Thickness	2	24,272.817	12,136.409	310.468	<0.001
LCU	2	8374.910	4187.455	107.121	<0.001
Curing Time × Overlay Thickness	6	1276.274	212.712	5.441	<0.001
Curing Time × LCU	6	1057.676	176.279	4.509	<0.001
Overlay Thickness × LCU	4	2026.656	506.664	12.961	<0.001
Curing Time × Overlay Thickness × LCU	12	1529.133	127.428	3.260	<0.001
Residual	288	11,258.136	39.091		
Total	323	149,846.094	463.920		
**AUR Group**					
**Factor**	**Degrees of Freedom**	**Sum of Squares**	**Mean Squares**	**F**	***p***
Curing Time	3	52,042.761	17,347.587	348.362	<0.001
Overlay Thickness	2	9791.130	4895.565	98.309	<0.001
LCU	2	6106.842	3053.421	61.317	<0.001
Curing Time × Overlay Thickness	6	1146.312	191.052	3.837	0.001
Curing Time × LCU	6	1058.841	176.473	3.544	0.002
Overlay Thickness × LCU	4	336.654	84.163	1.690	0.152
Curing Time × Overlay Thickness × LCU	12	297.039	24.753	0.497	0.916
Residual	288	14,341.690	49.798		
Total	323	85,121.267	263.533		

**Table 4:** Mean Vickers Hardness (VH) numbers (Mpa) recorded in experimental and control groups. Numbers in brackets represent standard deviations.

**ENA Group (Optimum Micro-Hardness (OM) Reference Value Recorded in the Control Group = 85.1 (4.6) MPa)**												
	**Curing Time with Halogen Unit** **(600 mW/cm^2^)**				**Curing Time with LED Unit** **(800 mW/cm^2^)**				**Curing Time with LED Unit** **(1500 mW/cm^2^)**			
Overlay Thickness	10 s	20 s	40 s	80 s	10 s	20 s	40 s	80 s	10 s	20 s	40 s	80 s
3 mm	19.7 ^d^_1_ (2.8)	36.8 ^c^_1_ (7.6)	58.2 ^b^_1_ (5.0)	**69.4 ^a^_1_** **(6.7)**	30.4 ^d^_1_ (5.8)	37.3 ^c^_1_ (11.0)	64.4 ^b^_1_ (8.9)	**79.4 ^a^_1_** **(8.7)**	43.6 ^d^_1_ (4.8)	56.5 ^c^_1_ (6.1)	**71.0 ^b^_1_** **(2.8)**	**82.3 ^a^_1_** **(4.0)**
4 mm	13.1 ^d^_2_ (2.2)	24.5 ^c^_2_ (5.5)	44.9 ^b^_2_ (7.4)	55.2 ^a^_2_ (2.4)	14.9 ^d^_2_ (3.8)	37.2 ^c^_1_ (8.8)	56.2 ^b^_2_ (7.2)	**72.4 ^a^_2_** **(3.0)**	19.3 ^d^_2_ (5.1)	30.2 ^c^_2_ (7.0)	49.3 ^b^_2_ (7.2)	**71.0 ^a^_2_** **(4.0)**
5 mm	8.1 ^d^_2_ (4.8)	21.7 ^c^_2_ (6.4)	27.4 ^b^_3_ (7.3)	47.2 ^a^_3_ (8.6)	13.5 ^d^_2_ (5.5)	29.4 ^c^_1_ (7.2)	45.6 ^b^_3_ (5.3)	61.9 ^a^_3_ (7.4)	18.2 ^d^_2_ (8.3)	25.5 ^c^_2_ (3.4)	37.9 ^b^_3_ (4.4)	61.3 ^a^_3_ (5.9)
**AUR Group (Optimum Micro-Hardness (OM) Reference Value Recorded in the Control Group = 63.6 (2.3) MPa)**												
	**Curing Time with Halogen Unit** **(600 mW/cm^2^)**				**Curing Time with LED Unit** **(800 mW/cm^2^)**				**Curing Time with LED Unit** **(1500 mW/cm^2^)**			
Overlay Thickness	10 s	20 s	40 s	80 s	10 s	20 s	40 s	80 s	10 s	20 s	40 s	80 s
3 mm	26.2 ^d^_1_ (4.2)	39.7 ^c^_1_ (5.7)	48.2 ^b^_1_ (4.3)	**58.1 ^a^_1_** **(3.0)**	26.7 ^d^_1_ (22.0)	41.3 ^c^_1_ (3.2)	**51.1 ^b^_1_** **(4.6)**	**59.1 ^a^_1_** **(5.3)**	43.5 ^d^_1_ (4.0)	**51.0 ^c^_1_** **(3.6)**	**59.4 ^b^_1_** **(4.2)**	**62.6 ^a^_1_** **(3.2)**
4 mm	16.8 ^d^_2_ (21.3)	29.2 ^c^_2_ (5.5)	43.4 ^b^_2_ (4.5)	**51.6 ^a^_2_** **(8.9)**	19.2 ^d^_2_ (4.8)	34.8 ^c^_2_ (10.0)	47.2 ^b^_2_ (4.9)	**57.0 ^a^_2_** **(2.6)**	27.3 ^d^_2_ (2.8)	39.6 ^c^_2_ (2.4)	**49.8 ^b^_2_** **(4.1)**	**57.3 ^a^_2_** **(4.1)**
5 mm	8.2 ^d^_3_ (4.8)	20.4 ^c^_3_ (6.6)	34.1 ^b^_3_ (5.7)	50.8 ^a^_3_ (3.6)	10.7 ^d^_3_ (2.1)	24.0 ^c^_3_ (3.4)	43.6 ^b^_3_ (4.8)	**52.1 ^a^_3_** **(7.0)**	22.3 ^d^_2_ (5.0)	35.8 ^c^_2_ (9.4)	49.1 ^b^_2_ (4.3)	**54.5 ^a^_2_** **(2.2)**

Within each different curing unit, the same superscript letters indicate no statistically significant differences among the levels of the factor “Curing Time” (reading horizontally). The same subscript numbers indicate no statistically significant differences among the levels of the factor “Overlay Thickness” (reading vertically). Numbers in bold represent mean VH values above 80% of each respective OM reference value, recorded in the heat-cured control groups.

## Data Availability

The data presented in this study are available on request from the corresponding author.
